# Impact of the COVID-19 pandemic on ovarian tissue cryopreservation planning in the Bologna clinical center

**DOI:** 10.3389/fonc.2022.1069274

**Published:** 2022-12-15

**Authors:** Valentina Magnani, Rossella Vicenti, Raffaella Fabbri, Roberto Paradisi, Lucia De Meis, Diego Raimondo, Paolo Casadio, Renato Seracchioli

**Affiliations:** ^1^ Department of Medical and Surgical Sciences, University of Bologna, Bologna, Italy; ^2^ Division of Gynaecology and Human Reproduction Physiopathology, IRCCS Azienda Ospedaliero-Universitaria di Bologna, Bologna, Italy

**Keywords:** ovarian tissue cryopreservation, oncofertility counselling, fertility preservation, COVID-19, oncological patients

## Abstract

**Introduction:**

Treatment of patients with COVID-19 has been a priority by competing with the treatment of any other disease due to limited hospital resources. The current pandemic situation has focused the attention of healthcare providers around the world away from all other non-emergency health problems, including oncofertility. The aim of the study was to evaluate the repercussions of the COVID-19 pandemic on the activity levels of ovarian tissue cryopreservation (OTC) in the our center.

**Methods:**

The study analyzed the number of patients treated for OTC in our center during three periods: pre-pandemic period: March 2019-February 2020, pandemic period: March 2020-February 2021 and post-pandemic period: March 2021-February 2022.

**Results:**

In our center routine hospital operation was completely reorganized, allowing only urgent interventions. Continuing to urgently preserve fertility during the pandemic required rapid changes to our standard practices for the care of these vulnerable patients. Despite the modifications, there was no difference in the number of OTC performed among the periods analyzed. Similarly, the number of patients who did not perform OTC was the same over the three years analyzed.

**Discussion:**

Despite the local and national restructuring of care to conserve resources and protect the community, it is significant to continue offering fertility-sparing treatment to cancer patients. This emphasis on the importance of preserving fertility despite the pandemic further highlights the essential and urgent nature of this procedure.

## Introduction

The COVID-19 pandemic posed unprecedented challenges to healthcare systems across the globe. Treatment of patients with COVID-19 has been a priority by competing with the treatment of any other disease due to limited hospital resources. Another crucial factor to consider is the fact that patients exhibited fear-related reluctance behaviors by postponing or even avoiding clinic visits for newly diagnosed symptoms or diseases. The main challenge for healthcare professionals around the world was to create resources to treat patients with COVID-19, while keeping the treatment of other serious diseases, such as cancer, unchanged.

Italy was the first Western country to be severely affected by the COVID-19 pandemic, consequently also the first to reorganize its health system to cope with the pandemic. The dramatic impact that the rampant infection created, has led to major changes affecting the prevention, diagnosis, and treatment of all diseases.

Screening programs were suspended in most regions between March and June 2020, preventing detection of early diagnoses, and are not resumed optimally in the following months. Likewise, surgical disciplines were also affected by the effects of the pandemic, leading to the indefinite suspension of elective surgery. In particular the number of surgeries decreased during the pandemic, with the time between multidisciplinary discussion and surgery doubled (1).

In addition, there is a particular area in which a total cessation of activity would have a definitive deleterious effect: that of fertility-sparing treatment in cancer patients undergoing highly gonadotoxic treatments (2–4).

Our center continued fertility-sparing treatment in cancer patients, but as cancer patients are at increased risk of severe morbidity or mortality from COVID-19 (5), our approach to fertility-sparing treatment was modified for safety reasons by minimizing in-office visits and monitoring.

The aim of the study was to evaluate the repercussions of the COVID-19 pandemic on the activity levels of ovarian tissue cryopreservation (OTC) in the our center, considering the periods before, during and after lockdown. We also evaluated the number of patients requiring highly gonadotoxic treatment who permanently lost the chance to undergo OTC, because it was materially impossible in the local health setting.

## Materials and methods

### Study design and patients

The study analyzed the number of patients treated for OTC in our center during three periods: pre-pandemic period: March 2019-February 2020; pandemic period: March 2020-February 2021 and post-pandemic period: March 2021-February 2022. The number of patients who lost the chance to undergo fertility-sparing treatment during the three periods was also evaluated. The OTC procedure and patient data collection were approved by our local Ethics Committee (N. 74/2001/O). An informed written consent was signed by all patients, and, in the case of minors, by their parents. The study was conducted in accordance with the Declaration of Helsinki.

This retrospective study includes girls and women aged a few months to 38 years who were at risk of premature ovarian failure for an oncological, hematological, or other pathology (genetic or autoimmune diseases) and therefore intended to preserve this function. Inclusion criteria for enrollment were: no previous pelvic surgery, no perimenopausal conditions identified by FSH and LH levels, no abnormalities in ovarian morphology with age-matched normal sonographic markers, no evidence of endocrine/metabolic or systemic diseases (6).

### Fertility-sparing program: OTC

The fertility-sparing program developed by our center aims to provide patients with accurate and complete advice on fertility-sparing and the assessment of ovarian function, taking all possible measures to avoid delays in the start of treatments.

The fertility-sparing program is based on a network of oncologists, oncohaematologists, pediatricians from hospitals in numerous regions of Italy. Upon diagnosis, healthcare requires an appointment at our center *via* dedicated phone or fax number and e-mail address to ensure quick access to the program. Once contact was established with the referring physician, the patient receives an appointment within a maximum of 7 days.

To ensure greater safety of the entire course of OTC procedure, patients were evaluated by the specialist team of our center, composed of gynecologists, endocrinologists, biologists, pediatric surgeons, anesthesiologists, nurses, and psychologists. Within a period of 24-36 hours before OTC, all patients, on a random day of the their menstrual cycle, underwent hormonal assay for follicle stimulating hormone (FSH), luteinizing hormone (LH), prolactin (PRL), Estradiol (E2), 17- hydroxyprogesterone (17-P), inhibin B (In-B), anti müllerian hormone (AMH) and ultrasound investigation for some ovarian markers, i.e. mean ovarian volume, antral follicle count (AFC), presence of corpus luteum or dominant follicle (6).

Surgery is scheduled within 24-48 hours of the investigation. A large ovarian biopsy from one ovary, or both ovaries in exceptional cases, was obtained by laparoscopy, in day surgery under general anesthesia, taking care to avoid damage to the ovarian tissue (with the use of Endobag), according to the technique of Paradisi et al. (7). To ensure safe surgery for pediatric patients, the surgery was performed according to the procedure of Lima et al. (8). The harvested tissue was frozen slowly according to the protocol described by Fabbri et al. (9). All patients were discharged after 24-48 hours and follow-ups were planned according to patient availability.

### Covid-19 modification

During the 2020 COVID-19 pandemic surge, fertility-sparing program was modified to limit possible COVID exposure to patients and staff and to optimize the use of available resources. All patients had SARS-CoV-2 pharyngo-nasal swab before entering in hospital and negativity to SARS-CoV-2 became an additional inclusion criterion for OTC procedure. Only in cases of immediate onset of chemotherapy and the inability to perform other fertility-sparing techniques OTC was offered to SARS-CoV-2 pharyngo-nasal swab positive patients. Patients analyses and surgery were performed on the same day. During emergency period no follow-up (gynecological check-up and hormonal tests) for patients were organized.

Social distancing measures thorough the office included significant changes to the waiting room (distance between chairs, plexiglass dividers between staff and patients, and distance between patients). Sanitation protocols were used to ensure all surfaces in the room were thoroughly cleaned between patients. Personal protective equipment was used by all staff.

### Covid-19 detection in ovarian tissue

For each SARS-CoV-2 pharyngo-nasal swab positive patient, two samples from medullary and cortical regions of the retrieved ovarian tissue were cut and sent together with peritoneal swabs and washings to the virology department for SARS-CoV-2 RNA detection as previously reported by Seracchioli et al. (10).

Briefly, extraction and purification of nucleic acids were performed using ELITe InGenius^®^ instrument with the ELITe InGenius SP 200 (ELITechGroup, Puteaux, France). For the qualitative detection of SARS-CoV-2 the GeneFinderTM COVID-19 PLUS RealAmp Kit (OSANGHealthcare, Gyeonggi, South Korea) was used on the ELITe InGenius^®^ instrument. The kit detects RNA-dependent RNApolymerase (RdRp), envelope (E), and nucleocapsid (N) genes of SARS-CoV-2.

### Statistical analysis

Differences among groups were evaluated in univariate analysis by means of non-parametric tests. Confidence intervals were estimated at 95% level, and all tests were two-sided, accepting *p* <.05 a indicating a statistically significant difference.

## Results

The data collected from March 2019 to March 2022 highlight how Covid19 pandemic did not delay the organization of the OTC procedure, the median time for laparoscopic surgery was 5 days (range 2 - 10 days) in the analyzed years. **Table 1** reports the number of patients consulted at our center. Three hundred five patients aged 1-38 years (median age 27.3 years) were selected. Of these, 70 (23%) were pediatrics and adolescent girls (≤ 17 years) and 235 (77%) were young and adult women (18 - 38 years). A total of 234 (58 pediatric and 176 adult women) cryopreserved ovarian tissue while 71 (12 pediatric and 59 adult women) lost the chance to manage their fertility-sparing.

**Table 1:** Number of patients counseled at Bologna clinical center from March 2019 to March 2022.

	MARCH 2019FEBRUARY 2020	MARCH 2020FEBRUARY 2021	MARCH 2021FEBRUARY 2022
**PEDIATRIC PATIENTS**			
**Ovarian tissue cryopreservation**	**25**	**16**	**17**
**NO Ovarian tissue cryopreservation**	**8**	**2**	**2**
**CONSULTANCIES**	**33**	**18**	**19**
**ADULT PATIENTS**			
**Ovarian tissue cryopreservation**	**58**	**53**	**65**
**NO Ovarian tissue cryopreservation**	**16**	**19**	**24**
**CONSULTANCIES**	**74**	**72**	**89**
**TOTAL**			
**Ovarian tissue cryopreservation**	**83**	**69**	**82**
**NO Ovarian tissue cryopreservation**	**24**	**21**	**26**
**CONSULTANCIES**	**107**	**90**	**108**

Considering the number of procedures over the years, no differences were observed comparing the number of OTC procedure performed in the analyzed period. Similarly, the number of patients who did not perform OTC was the same over the three years analyzed.

Baseline patient characteristics from March 2019 to March 2022 are presented in **Table 2**. Medical diagnoses and indications for urgent OTC from 2019 to 2021 were similarly divided between benign and malignant diseases (**Table 2**).

**Table 2:** Baseline characteristics of the patients from March 2019 to March 2022.

	MARCH 2019FEBRUARY 2020	MARCH 2020FEBRUARY 2021	MARCH 2021FEBRUARY 2022
**BASELINE CHARACTERISTICS OF PATIENTS**			
**Age (years)**	21,54	25,32	24,16
**FSH (mIU/ml)**	8,4	6,5	7,9
**LH (mIU/ml)**	15,7	12,3	8,2
**PRL (ng/ml)**	7,5	10,1	9,4
**E2 (pg/ml)**	180,2	243,9	87,3
**AMH (ng/ml)**	2,5	1,8	3,1
**AFC**	8	11	9
**DIAGNOSIS OF ADULT PATIENTS**			
**Breast cancer**	11	8	14
**Hodgkin Lymphoma** **Non-Hodgkin Lymphoma**	38	34	33
**Sarcoma**	3	1	3
**Thalassemia**	0	3	3
**Leukemia** **Myelofibrosis**	1	4	1
**Brain tumor**	2	0	4
**Other malignancy**	3	3	7
**Total**	**58**	**53**	**65**
**DIAGNOSIS OF PEDIATRIC PATIENTS**			
**Hodgkin Lymphoma** **Non-Hodgkin Lymphoma**	10	7	4
**Sarcoma**	6	2	5
**Brain tumor**	1	1	4
**Leukemia** **Myelofibrosis**	0	0	2
**Thalassemia**	5	1	1
**Other malignancy**	3	5	1
**Total**	**25**	**16**	**17**

Analyzing the 2020 OTC procedure in detail, 20 (29%) were performed despite unknown COVID status before the start of the routine SARS-CoV-2 pharyngo-nasal swab test in April 2020. As of April 2020, all patients treated were negative for SARS-CoV-2, except for three patients with Hodgkin lymphoma: two of them underwent OCT after recovery as the disease did not require immediate chemotherapy. The remaining patient, with SARS-CoV-2 positive pharyngo-nasal swab, underwent OTC during the infection period due to the immediate onset of chemotherapy and the inability to perform other fertility-sparing techniques. Two SARS-CoV-2 positive patients refused the OTC procedure. No patients contracted SARS-CoV-2 during OTC procedure.

**Table 3** summarized the medical diagnoses of the patients who did not performed OTC from March 2019 to March 2022. The reasons for the OTC failure were the same every year. The main contraindication to OTC was lack of indication for cryopreservation (i.e., age limit or delay in the start of treatments), surgical contraindication, fear of surgery, personal choice (i.e., fear for SARS-CoV-2 infection) (**Table 4**).

**Table 3:** Medical diagnoses of the patients that did not performed OTC procedure from March 2019 to March 2022.

	MARCH 2019FEBRUARY 2020	MARCH 2020FEBRUARY 2021	MARCH 2021FEBRUARY 2022	MARCH 2019FEBRUARY 2020	MARCH 2020FEBRUARY 2021	MARCH 2021FEBRUARY 2022
	**ADULT PATIENTS**			**PEDIATRIC PATIENTS**		
**Breast cancer**	2	3	5			
**Lymphomas**	10	14	10	2		
**Sarcomas**			1	1		1
**Thalassemia**	1			1	1	
**Leukemia** **Myelofibrosis**	1		2	1		
**Brain tumor**				1		
**Other malignancy**	2	4	6	2	1	1
**Total**	**16**	**19**	**24**	**8**	**2**	**2**

**Table 4:** Reasons for the OTC failure from March 2019 to March 2022.

	MARCH 2019FEBRUARY 2020	MARCH 2020FEBRUARY 2021	MARCH 2021FEBRUARY 2022	MARCH 2019FEBRUARY 2020	MARCH 2020FEBRUARY 2021	MARCH 2021FEBRUARY 2022
	**ADULT PATIENTS**			**PEDIATRIC PATIENTS**		
**Lack of cryopreservation indication**	5	5	11	4	1	1
**Surgical controindication**	4	3	3	2		1
**Fear of surgery**	7	7	9	2	1	
**Personal choice**		4	1			
**Total**	**16**	**19**	**24**	**8**	**2**	**2**

## Discussion

Rapid community transmission and relatively high mortality rates with Covid-19 are relatively unique features and have led to an unprecedented global health crisis. The current pandemic situation has focused the attention of healthcare providers around the world away from all other non-emergency health problems, including oncofertility (11).

Any newly diagnosed cancer patient, who may wish to have children in the future should be informed about their fertility-sparing options by a specialist in reproduction field including gynecologists, endocrinologists, biologists, pediatric surgeons, anesthesiologists, nursing staff, and psychologists (12). If the person with cancer wishes to implement fertility-sparing measures, close interdisciplinary cooperation between healthcare providers is in the best interest. The Covid19 Task Force of The American Society for Reproductive Medicine (ASRM) and The Society for Assisted Reproduction Technology have issued special recommendations on preserving fertility, adapted to the Covid-19 pandemic situation (13, 14). The guidelines emphasize that fertility-sparing measures must be considered an emergency treatment to be carried out without delay, taking into account mandatory hygiene measures (15).

Due to the increasing importance of fertility protection measures, it is important to provide techniques that preserve and restore fertility even in times of pandemic. In our center routine hospital operation was completely reorganized, allowing only urgent interventions. Continuing to urgently preserve fertility during the pandemic required rapid changes to our standard practices for the care of these vulnerable patients. Despite the modifications, there was no difference in the number of OTC performed among the periods analyzed.

The presence of SARS-CoV-2 has been reported in several tissue types, including respiratory, ileum, heart, and kidney, for which established COVID-19 symptoms are also present (16). Regarding the reproductive field, although there is controversial evidence on the presence of SARSCoV-2 in seminal plasma of COVID-19-infected or recovering patients, so far there are no reports demonstrating its presence in female reproductive cells and tissues (17). A potential susceptibility of ovarian tissue to SARSCoV-2 infection has only been hypothesized *in vitro*, while there are no *in-vivo* studies on its presence in ovarian tissue of SARS-CoV-2 positive women (18). We demonstrated the absence of viral RNA and the negative molecular analysis of ovarian tissue in two women infected with SARS-CoV-2 before cryopreservation (10). Moreover COVID-19 infection does not appear to directly affect the ovarian follicular reserve and therefore fertility, at least in asymptomatic patients. There is no information on the possibility of transplanting the ovarian tissue retrieved from the SARS-CoV-2 positive patient. In the literature, few studies have been published on the evaluation of the short-term outcomes in patients who received various transplant from SARS-CoV-2 positive donors. Although the data is very limited, they reported that the recipients receiving products from these donors does not adversely affect engraftment or survival (19, 20). Despite these findings, the drafting of official guidelines of the national transplant center will be necessary for planning future cryopreserved ovarian tissue transplant in patient SARS-CoV-2 positive at the time of ovarian tissue retrieval.

A relevant risk in cryopreservation is the potential transmission of pathogens during preparation and cryopreservation procedures. Although, the transmission of pathogens in samples stored in fertility cryobanks has never been reported (21), the cryo-resistance of SARS-CoV-2 is extremely high (22). So it is important to understand and mitigate these risks through safety and precautionary measures. Some of these include testing for SARSCoV-2 before OTC, sanitary cryostorage protocols and efficient tissue washing during cryopreservation, which will help reduce the risk of disease transmission (17).

## Conclusion

Summarizing, given the lasting impacts of COVID-19 and the continuing recurrence of new variants, many of our protocols have remained in place. The risk of SARSCoV- 2 infection for vulnerable populations is likely to persist in the near future, and clinics need to be prepared to rotate and change care for other urgent/emerging indications (23).

The COVID-19 pandemic led to massive disruptions to medical care worldwide. Despite the local and national restructuring of care to conserve resources and protect the community, it is significant to continue offering fertility-sparing treatment to cancer patients. This emphasis on the importance of preserving fertility despite the pandemic further highlights the essential and urgent nature of this procedure.

## Data availability statement

The raw data supporting the conclusions of this article will be made available by the authors, without undue reservation.

## Ethics statement

The studies involving human participants were reviewed and approved by 74/2001/O. Written informed consent to participate in this study was provided by the participants’ legal guardian/next of kin.

## Author contributions

VM, RS: planned the study. DR, PC: performed ovarian tissue retrieved. RV, VM: cryopreserved and thawed ovarian tissue. RV, VM, RP, RF: nalyzed and collected data, drafted the paper. LD: performed gynecologic evaluation of the patient. All authors contributed to the article and approved the submitted version.
